# Diseases of the Heart and Circulation

**Published:** 1904-07-09

**Authors:** 


*(To be continued.)*

### DISEASES OF THE HEART AND CIRCULATION.

**Anatomy.**—In the Hunterian lectures of Keith[1] a valuable account is given of researches in the development of the heart which have bearing on some as yet unexplained clinical phenomena.

**Closure of the Venæ Cavæ.**—Normally, in lower vertebrates, and during the third, fourth, and fifth months of development of man, no regurgitation takes place from the right auricle during its systole owing to the presence of valves (a right and a left) guarding the orifice of the sinus venosus into the auricle. These valves disappear in succeeding months, and the sinus is taken up into and forms part of the auricle. The musculature at the base of the left valve atrophies whilst that of the right valve increases in size forming the right tænia terminalis. During the systole of the auricle the orifice of the superior vena cava is closed by the muscular bands surrounding its orifice, aided by the right tænia terminalis. The orifice of the inferior vena cava is closed partly by muscular, partly by mechanical means. These measures are so effectual that, in health, the pulsation in the large veins is due merely to a damming back of the blood in them, there being no actual regurgitation. In disease actual regurgitation occurs.

**Adherent Pericarditis.**—In health the heart has distinct respiratory movements owing to its being firmly fixed to the roots of the lungs, in inspiration the base of the heart moving forwards and downwards. This is due to the fact that the apex of the lung is the fixed point from which the inspiratory movement of the lung takes place, and from it expansion proceeds in a forward, downward, and outward direction. Now, though mere adhesion of the heart to the pericardium does not interfere with these respiratory movements, when adhesions fix the pericardium to the chest wall these movements become impossible. The patient then developes a type of respiration which can be performed without moving the roots of the lungs, *i.e.* by expanding his chest in a lateral direction, the central part of the diaphragm and its crura taking no part.

**Movements of the Right Auricle.**—Physiologists have shown that after the systole of the right auricle is over there appears in that cavity a negative pressure lasting during the opening two-thirds of the systole of the ventricles. This is of great importance in drawing the venous blood into the heart, but its mechanism is ill understood. It is, in reality, due to an active expansion of the right auricle by the contraction of the right ventricular wall. During systole the apex of the ventricles remains fixed and the base of the right ventricle moves towards the apex of the heart. The auricular cavity thus increases in dimensions.

**Fixation of the Apex.**—In reptiles the cardiac apex is fixed by a ligament, but in birds and mammals this end is accomplished by the peculiar structure of the left ventricle. Its wall consists of three layers of muscle, an inner and an outer longitudinal layer with a middle spiral or circular layer. All three layers meet at the apex. During systole the apex becomes the seat at which two forces act in opposite directions, the thick layer of circular fibres would drive it outwards, whilst the longitudinal layers act in the opposite direction and draw it inwards. The two forces are equally balanced in expelling the ventricular column of blood, and hence the comparatively stationary position of the apex.

**Closure of the Foramen Ovale.**—Before birth about two-thirds of the blood entering the right auricle passes through the foramen ovale. It is directed thither from the inferior vena cava by the active contraction of the limbic muscular bands which make up the annulus ovalis; these draw up the inferior vena cava towards the foramen ovale, so that the arterial blood from the placenta passes into the right auricle. With the first descent of the diaphragm in respiration the direction of these limbic bands is altered, becoming more vertical in direction, owing to the termination of the inferior vena cava, firmly fixed in the diaphragm, being drawn downwards. The origin of the bands in the interauricular septum is thus drawn towards the inferior vena cava and helps to close the foramen ovale. The fall of pressure in the right ventricle and the rise in the left auricle are the other conditions which lead to its closure.

**Closure of the Ductus Arteriosus.**—Before birth, owing to the pressure in the pulmonary artery being greater than in the aorta, the wall of the ductus arteriosus is round in shape and projects within the aorta, giving that vessel a crescentic shape. At birth, owing to the opening up of the pulmonary circulation and closure of the foramen ovale, the pressure in the aorta becomes at least twice that in the ductus, the lumen of which is consequently diminished. A second factor in closing the ductus is that, whereas before birth the ductus is horizontal in position, at birth the descent of the diaphragm drags down the whole pericardium and its contents. The arch of the aorta does not descend, owing to its being bound to the neck by the cervical fascia. Hence a strain is thrown on the ductus and assists in closing it.

[1] Keith, Lancet, Feb. 27, March 5 and 12.

